# Barriers to Buruli ulcer treatment completion in the Ashanti and Central Regions, Ghana

**DOI:** 10.1371/journal.pntd.0008369

**Published:** 2020-05-26

**Authors:** Shelui Collinson, Venus N. B. Frimpong, Bernadette Agbavor, Bethany Montgomery, Michael Oppong, Michael Frimpong, Yaw A. Amoako, Michael Marks, Richard O. Phillips

**Affiliations:** 1 Clinical Research Department, London School of Hygiene and Tropical Medicine, London, United Kingdom; 2 Kumasi Centre for Collaborative Research, Kwame Nkrumah University of Science and Technology, Kumasi, Ghana; 3 Hospital for Tropical Diseases, London, United Kingdom; Barwon Health, AUSTRALIA

## Abstract

**Background:**

Buruli ulcer is a chronic ulcerating skin condition, with the highest burden found in Central and West Africa where it disproportionately affects the most vulnerable populations. Treatment is demanding, comprising eight-weeks of daily antibiotics, regular wound care and possible surgical intervention. Treatment completion is key to optimising outcomes, however the degree of and barriers to this are not well understood. Recent change from injectable treatment (SR8) to oral treatment (CR8) has made it feasible to further decentralise care, potentially improving treatment access and completion. However, the impact of this and of other demographic and clinical influences on treatment completion must be explored first to ensure appropriate models of care are developed.

**Methodology/Principal findings:**

A retrospective clinical notes review and secondary data analysis of records from patients diagnosed between 1 January 2006–31 December 2018 at four district hospital clinics in the Ashanti and Central Regions, Ghana. Univariable analyses and multivariable logistic regression were performed to assess the association between explanatory variables and treatment completion.

There were 931 patient episodes across the four clinics with overall treatment completion of 84.4%. CR8 was associated with higher treatment completion compared to SR8 (OR 4.1, P = 0.001). There was no statistically significant association found between distance from patient residence to clinic and treatment completion.

**Conclusions/Significance:**

Improved treatment completion with CR8 supports its use as first line therapy and may enable decentralisation to fully community-based care. We did not find an association between distance to care and treatment completion, though analyses were limited by data availability. However, we did find evidence that distance to care continues to be associated with more severe forms of disease, which may reflect the higher costs of accessing care and lower awareness of the condition the further a patient lives. Decentralised care must therefore also continue to support community engagement and active outreach to identify cases early.

## Author summary

Buruli ulcer (BU) is a chronic ulcerating tropical skin disease known to particularly affect vulnerable populations. Without early detection and effective treatment it can lead to disfigurement, disability and stigma. In order to improve outcomes, we need to understand what factors prevent patients from accessing and completing treatment, however these factors are often not well understood. Factors considered to potentially affect treatment completion include access to care and type of treatment. In this study we analysed data available from clinical records of patients treated in Ghana to identify whether type of treatment and common patient characteristics were associated with treatment completion. We found that treatment completion was higher in patients who took a newly introduced oral treatment compared to those who took the traditional injectable treatment. We did not find a difference in treatment completion between patients living close to the clinic and those living further away, however we found that those living further were more likely to present with more advanced disease. The results from this study suggest that management for patients living far from care needs to be improved. The newly recommended oral treatment makes it feasible to provide care away from health centres and the improved treatment completion seen in this study supports its use. However, further research should be conducted to determine how fully community based care can best be provided.

## Introduction

Buruli ulcer (BU) is a chronic, ulcerating skin condition resulting from infection with *Mycobacterium ulcerans* [1]. Classified as a neglected tropical disease (NTD), it disproportionately affects some of the world’s poorest populations [2]. The distribution of BU is geographically focal [3] and whilst it is known to be prevalent in 33 countries, including parts of Asia, South America and the Western Pacific, the highest burden is in Central and West Africa [1]. In Ghana, BU is one of the most common skin NTDs [4], and in 2018 it had the highest number of reported cases globally [5], though this may be due, in part, to efforts to improve early case detection [6]. Despite this, BU is known to be considerably underreported across endemic countries, owing to factors including the stigma surrounding skin conditions, logistical and financial difficulties accessing care and problems with recognition and diagnosis of the condition [3]. Clinical manifestations range from small nodules, plaques or ulcers to larger lesions which can extend to the bone or affect critical sites including the head and genitalia [7]. With the potential to cause severe long-term morbidity, prompt and effective management is essential [8].

Completion of BU treatment is deemed necessary in order to ensure adequate wound healing and prevent recurrence [7,9], however the degree and characteristics of BU treatment completion in West Africa have not been extensively researched. A study in Ghana suggested that treatment was only fully completed in 46% of cases between 2008–2012 [10] but other studies have reported much higher rates of treatment completion in the region of 94–98% [11,12]. Given such wide discrepancies further work is needed to understand factors associated with adherence and successful BU treatment completion.

Possible barriers to treatment completion have included adverse effects of the treatment itself and the direct and indirect costs and accessibility of care. Until recently, patients required eight weeks of daily antibiotics with rifampicin and the injectable streptomycin (SR8) [7]. Both pain from injections and the toxicity of streptomycin, with its risks of hearing loss and renal impairment, have been regarded as key issues affecting the management of BU [13,14]. Recently, a fully oral regimen of clarithromycin and rifampicin (CR8) has been proven equally efficacious and is now recommended by WHO [1,15,16]. Financial and logistical difficulties in accessing care have also been implicated as key factors determining treatment completion [10,13]. An economic analysis of the cost of outpatient care for BU in Ghana indicated the potential for it to place catastrophic financial demands on patients: the total household costs of treating one case, including the costs required for transportation to access care and the indirect costs arising from productivity loss were found to constitute almost half of the annual household income [17]. Recent studies indicate that whilst health education has helped improve understanding of the condition in Ghana, many patients will still seek local herbal treatment due to concerns regarding the costs of transportation and the accessibility of BU clinics [18]. With the issues of cost and accessibility of care delaying or preventing appropriate care seeking, it is feasible that they may also affect treatment completion. The new fully oral treatment regimen may improve treatment adherence both through increased tolerability compared to the injectable regimen but also because it reduces the need for healthcare facility visits to receive daily injections and is therefore associated with reduced indirect patient costs.

A number of mechanisms to support treatment completion have been suggested and are particularly aimed at overcoming logistical and financial barriers patients face. Provision of free transportation to clinics, reducing both the direct transportation costs and, with its increased convenience, the opportunity costs of spending time away from employment or school, has been suggested [19]. The notion of bringing care closer to patients has also been proposed and is supported by the improved treatment retention and increased likelihood of treatment success seen through decentralisation of care in conditions such as tuberculosis (TB) and HIV [20–23]. There has been increasing decentralisation of care for BU. Whilst surgery used to be the mainstay of management, the introduction of an antibiotic regimen in 2004 [24] has meant that now only more extensive BU lesions require surgical management [7]. Furthermore, with the introduction of the oral antibiotic regimen patients do not need to see a health worker daily to receive antibiotic injections, but rather at less frequent intervals to collect a supply of oral antibiotics [10,25]. Whilst patients still require regular, often daily, wound care [1], a study in Benin showed that if this can be provided locally, less severe lesions can be fully treated in the community, thus reducing the fear and costs associated with attending BU clinics [21]. Community-based treatment of BU is also likely to be mutually supportive of current activities aimed at early case-detection: it is likely to support the use of community-based volunteers to increase awareness and early referral of cases to BU facilities [20] and by diagnosing BU at an early stage, cases will be more amenable to community-based management.

There are concerns that moving to a fully community-based model and thereby removing any direct observation of therapy (DOT) could reduce treatment completion [10]. The use of DOT for similarly demanding treatment schedules, such as TB management, has shown some positive results; a recent meta-analysis found that DOT was associated with increased rates of TB treatment success compared to unobserved therapy, although the results for treatment completion and relapse were inconclusive [26]. Mechanisms have, however, been suggested to prevent a move away from clinic contact affecting treatment completion, including appointment reminders for regular defaulters and education for patients on treatment and side effects [11]. For less complex cases, clinical assessments could be performed locally by community health workers, thus reducing the burden of care further [25].

A better understanding of the barriers to BU treatment completion must be developed to inform changes in the management and delivery of care for BU. The extent to which both ongoing barriers in access to treatment and the introduction of the fully oral treatment have impacted treatment completion is not well understood. The aim of this study was to review the BU treatment completion rates in the Ashanti and Central Regions, Ghana, to explore factors associated with reduced treatment completion and assess the impact of the new oral treatment regimen on treatment completion.

## Methods

We conducted a retrospective study using data extracted from the standard WHO BU treatment forms (BU01 forms [27]). We included patients diagnosed between 1 January 2006 and 31 December 2018 in four clinics across the Ashanti Region (Agogo, Nkawie and Tepa clinics) and Central Region (Dunkwa clinic), which are estimated to have the highest prevalence of BU in Ghana [28]. Demographic and treatment information was supplemented by records from the WHO Buruli Ulcer Trial, which recruited patients between 2013 and 2017 [16]), where available.

All BU patients for whom clinical notes were available were included if treatment commenced and was either completed or assumed permanently defaulted (i.e. with either the patient documented as defaulted on the BU01 form or no further treatment doses recorded) within this window. Patients enrolled in the TOP trial (2007–2008) where a shorter, and therefore non-comparable, treatment regimen was studied, were excluded (n = 47).

Data extraction was undertaken at the Kumasi Centre for Collaborative Research in Tropical Medicine (KCCR) in Kumasi, which has established links with the four BU clinics. Usual practice is for three sets of BU01 forms to be filled in per patient, with one each for the patient, the health facility and the KCCR team. Where data was missing from the KCCR copy, the relevant treatment centres were visited where possible to assess the health facility version and the most complete copy used. Discrepancies were resolved by consensus amongst the study team.

### Statistical analysis

Data was cleaned in Excel and analysed in STATA 15 (StataCorp 2017, TX, USA). Preliminary data assessment was performed, including for the degree of missing data. If a variable had more than 10% of its data missing, the distribution of missing data across the main exposure categories was examined and disparities acknowledged in the interpretation of results.

Variables with fewer than ten outcomes in a stratum were regrouped where appropriate to increase stratum numbers for analysis. For the purpose of analysis, occupation was classified as farmer, pupil or other occupation; lesion location was classified as trunk, upper limb, lower limb and multiple/other; lesion form was classified as non-ulcer, ulcer and multiple/osteomyelitis; referral route was classified as village health volunteer, health worker and other and year of diagnosis was classified as 2006–10, 2011–14 and 2015–18. Univariable analysis was conducted, controlling for age and sex, which were considered *a priori* confounders. Variables with some evidence of association with treatment completion (p < 0.1) were included in the multivariable regression model, alongside the main explanatory variables ‘distance to clinic’ and ‘treatment regimen’. Lesion category, lesion form and duration of lesion were considered to be proxies for lesion severity and likely to stem from treatment accessibility. They were therefore thought to be on the causal pathway and so were not included in the multivariable regression model. Year of diagnosis was considered to be collinear with treatment regimen, with CR8 provided partially during 2013–16 as part of a WHO trial [16] and fully from 2017 onwards and was therefore excluded. The model was assessed for clustering at the facility level; as there was no evidence of clustering, a random effects model was not fitted.

### Variable determination

Distance to clinic was used as an indirect measure of treatment accessibility. Village locations were determined in ArcGIS using the Ghana Settlements data obtained from the Humanitarian Data Exchange [29]. Villages documented on BU01 forms were identified as those located closest to the clinic attended with the same name. If more than one potential location was identified and the correct one could not be determined by local staff, the village was not plotted. The point distance between the village and clinic attended was measured.

Treatment completion was determined in patients who had taken at least 95% of the full 56 days of treatment (i.e. with both antibiotics taken each day). A patient was considered to have not adequately completed treatment if they did not complete the course within a 70-day period, as such patients were expected to restart treatment.

### Ethics

The project was conducted as part of the research *Pathogenesis and Management of M*. *Ulcerans Disease (Buruli ulcer)*, which was approved by the Committee on Human Research and Publication Ethics of the School of Medical Sciences at the Kwame Nkrumah University of Science and Technology (Ref. CHRPE/AP/245/19).

## Results

A total of 978 BU patients were seen across the four clinics between 1 January 2006 and 31 December 2018. We excluded 47 patients who participated in the TOP trial, leaving 931 in the analysis (NB the denominator used throughout will vary due to missing data). There were three repeat presentations amongst the 931 patients. Overall 480 (52%) of the patients with sex documented were female and the median age was 14 (IQR 8–30). The majority of patients attended clinics in Agogo (49.1%) and Tepa (39.7%) (Table 1). Data on treatment completion was available for 676 patients (72.6%).

**Table 1:** Characteristics of the study population (total n = 931*).

Demographic characteristic	Details	
**Male sex**, n (%)	441 (47.9)	
Missing data	1.1%	
**Age**, years [median (IRQ)]	14 (8–30)	
Missing data	0.8%	
**Occupation**, n (%)		
Pupil	510 (59.0)	
Farmer	274 (31.7)	
Other^a^	80 (9.3)	
Missing data	7.2%	
**Year of diagnosis**, n (%)		
2006–10	301 (32.5)	
2011–14	453 (48.9)	
2015–18	173 (18.7)	
Missing data	0.4%	
**Clinic attended**, n (%)		
Agogo	457 (49.1)	
Dunkwa	58 (6.2)	
Nkawie	46 (4.9)	
Tepa	370 (39.7)	
Missing data	0%	
**Distance to clinic,** km [median (IQR)]	17 (6.2–24.2)	
Missing data (total)	39.6%	
Missing data (by regimen)	SR8 38%, CR8 44%	
**Referral route**, n (%) [OR for having advanced lesion** vs village health worker]		
Village health volunteer	294 (34.0)	
Health worker	373 (43.2) [OR 3.0, 95% CI 2.2–4.1, p < 0.001]	
Other^b^	197 (22.8) [OR 2.1, 95% CI 1.4–3.0, p < 0.001]	
Missing data	7.2%	
**Duration of lesion at diagnosis**, weeks [median (range; IQR)]	4 (0.5–1196; 2–8)	
Missing data	6.2%	
**Used traditional treatment**, n (%)	271 (31.7)	
Missing data	8.2%	
**Lesion Location**, n (%)		
Upper limb	349 (38.3)	
Lower limb	467 (51.2)	
Trunk	54 (5.9)	
Multiple/Other^c^	42 (4.6)	
Missing data	2%	
**Lesion form at diagnosis**, n (%) [% by distance to clinic; OR for living >20km from clinic vs those with non-ulcer]		
Non-ulcer	411 (44.9) [0- km: 35, 10- km: 41, 20+ km: 24]	
Ulcer	496 (51.3) [0- km: 21, 10- km: 34, 20+ km: 45; OR 2.6, 95% CI 1.8–3.7, p <0.001]	
Multiple/osteomyelitis	43 (3.8) [0- km: 10, 10- km: 33, 20+ km: 57; OR 4.2, 95% CI 1.7–10.4, p = 0.002]	
Missing data	1.7%	
**Lesion category at diagnosis**, n (%) [% by distance to clinic; OR for living >20km from clinic vs those with category I lesion]		
I	420 (47.1) [0- km: 31, 10- km: 44, 20+ km: 24]	
II	279 (31.3) [0- km: 23, 10- km: 35, 20+ km: 42; OR 2.3, 95% CI 1.5–3.4, p <0.001]	
III	192 (21.3) [0- km: 23, 10- km: 27, 20+ km: 50; OR 3.1, 95% CI 1.2–5.0, p <0.001]	
Missing data	4.3%	
**Treatment regimen taken**, n (%)		
SR8	698 (78.3)	
CR8	194 (21.8)	
Missing data	4.2%	
**Completed treatment**, n (%)	569 (84.4)	
Missing data (total)	27.4%	
Missing data (by regimen)	SR8 32%, CR8 6%	
Missing data (by distance)	0- km 35%, 10- km 28%, 20+ km 25%	

*Total n = 931, but all variable results are based on the data available for that variable

** ulcer, multiple lesions or osteomyelitis

**a:** Other occupation includes hairdresser, trader, miner, driver, tailor, teacher, unemployed, retired and all single-count occupations

**b:** Other referral route includes family member, former patient and self

**c:** other location includes eye, head and neck, breast, perineum and genitalia.

We found evidence that both lesion form and lesion category differed by distance to clinic. Patients with ulcerated lesions and multiple lesions or osteomyelitis were more likely to live over 20km from the clinic compared to patients with non-ulcer lesion forms (OR 2.6, 95% CI 1.8–3.7, p < 0.001 and OR 4.2, 95% CI 1.7–10.4, p = 0.002 respectively), whilst patients with category II and III lesions were also more likely to live over 20km from the clinic compared to patients with category I lesions (OR 2.3, 95% CI 1.5–3.4, p < 0.001 and OR 3.1, 95% CI 1.2–5.0, p < 0.001 respectively). We also found that patient referral pattern varied by lesion form. Patients referred by health workers were more likely to have more advanced lesion forms (ulcerated lesions and multiple lesions or osteomyelitis) compared to patients referred by village health volunteers (OR 3.0, 95% CI 2.2–4.1, p < 0.001) (Table 1).

Data on the distance to clinic was available for 562 (60.4%) patients. For these patients, the distance ranged from 0km (i.e. living in the village of the clinic) to 191.3km; the median distance to clinic was 17km (IQR 6.2–24.2km). There was no evidence of association between distance to the clinic and treatment completion with 79.8% of individuals living within 10km completing treatment compared to 87.4% of those living 10-20km from clinic (OR 1.51, 95% CI 0.72–3.14) and 87.3% of those living more than 20km from clinic (OR 1.36, 95% CI 0.66–2.83) (Table 2).

**Table 2:** Baseline variable distributions and their univariable associations with treatment completion, controlling for forced variables age and sex (total n = 931*).

Variable	No. (%) patients	No. (%**) completing treatment	Univariate OR (95% CI)	P value***
**Sex** (n = 921)				
Female Male	480 (52.1)	295 (84.5)	1	0.0582
	441 (47.9)	270 (84.4)	0.88 (0.57–1.35)	
**Age group** (years) (n = 924)				
0- 5- 10- 20- 30- 40- 50- 60+	81 (8.8)	48 (87.3)	1	0.0582
	200 (21.7)	126 (91.3)	1.56 (0.57–4.19)	
	286 (31.0)	187 (86.6)	0.98 (0.40–2.39)	
	114 (12.3)	61 (82.4)	0.67 (0.25–1.82)	
	94 (10.2)	55 (79.7)	0.56 (0.21–1.52)	
	60 (6.5)	36 (78.3)	0.51 (0.18–1.48)	
	42 (4.6)	27 (77.1)	0.48 (0.16–1.48)	
	47 (5.1)	26 (72.2)	0.38 (0.13–1.12)	
**Distance to clinic** (km) (n = 562)				
0- 10- 20+	153 (27.2)	79 (79.8)	1	
	210 (37.4)	132 (87.4)	1.51 (0.72–3.14)	
	199 (35.4)	130 (87.3)	1.36 (0.66–2.83)	0.0754
**Treatment regimen** (n = 892)				
SR8 CR8	698 (78.3)	385 (80.7)	1	
	194 (21.8)	170 (93.4)	4.27 (2.20–8.31)	<0.0001
**Clinic attended** (n = 931)				
Agogo Dunkwa Nkawie Tepa	457 (49.1)	238 (84.4)	1	0.1599
	58 (6.2)	39 (86.7)	1.28 (0.50–3.26)	
	46 (4.9)	29 (85.3)	1.28 (0.46–3.56)	
	370 (39.7)	263 (84.0)	1.09 (0.68–1.72)	
**Occupation** (n = 864)				
Farmer Pupil Other^a^	274 (31.7)	148 (74.4)	1	0.0054
	510 (59.0)	331 (88.7)	5.14 (1.78–14.89)	
	80 (9.3)	52 (85.3)	2.10 (0.95–4.64)	
**Traditional treatment use** (n = 855)				
No Yes	584 (68.3)	343 (82.3)	1	
	271 (31.7)	173 (87.4)	1.50 (0.91–2.48)	0.0607
**Lesion location** (912)				
Upper limb Lower limb Trunk Multiple/Other^b^	349 (38.3)	211 (84.7)	1	0.1129
	467 (51.2)	285 (83.3)	1.08 (0.68–1.71)	
	54 (5.9)	35 (85.4)	0.90 (0.35–2.36)	
	42 (4.6)	31 (93.9)	2.51 (0.57–11.06)	
**Referral route** (n = 864)				
Health worker Village health volunteer Other^c^	373 (43.2)	252 (83.4)	1	0.0444
	294 (34.0)	145 (82.4)	0.85 (0.51–1.41)	
	197 (22.8)	131 (87.3)	1.31 (0.73–2.35)	
**Duration of lesion** (weeks) (n = 873)				
0- 2- 3- 4- 5- 12- 52+	75 (8.6)	36 (83.7)	1	0.2589
	146 (16.7)	78 (83.9)	1.19 (0.43–3.27)	
	120 (13.8)	79 (83.2)	1.03 (0.38–2.78)	
	193 (22.1)	126 (86.9)	1.54 (0.59–4.09)	
	162 (18.6)	102 (80.3)	0.95 (0.37–2.44)	
	138 (15.8)	84 (84.0)	1.33 (0.49–3.59)	
	39 (4.5)	23 (79.3)	1.31 (0.36–4.79)	
**Year of diagnosis** (n = 927)				
2006–2010 2011–2014 2015–2018	301 (32.5)	109 (87.9)	1	
	453 (48.9)	320 (83.3)	0.62 (0.33–1.18)	
	173 (18.7)	138 (84.7)	0.87 (0.42–1.81)	0.0467
**Lesion form** (n = 915)				
Ulcer Non-ulcer Multiple/osteomyelitis	496 (51.3)	291 (83.9)	1	0.1139
	411 (44.9)	249 (85.6)	0.99 (0.63–1.58)	
	43 (3.8)	23 (76.7)	0.65 (0.25–1.73)	
**Lesion category** (n = 891)				
I II III	420 (47.1)	236 (85.5)	1	0.0911
	279 (31.3)	189 (84.0)	0.85 (0.51–1.41)	
	192 (21.3)	119 (83.8)	0.96 (0.54–1.71)	

*Total n = 931, but all variable results are based on the data available for that variable

**Denominator differs from the baseline data for that variable due to missing data for treatment completion.

***From LRT for association between variable and treatment completion, controlling for age and sex (age and sex are controlled for each other).

**a:** ‘Other’ occupation includes hairdresser, trader, miner, driver, tailor, teacher, unemployed, retired and all single-count occupations

**b:** ‘other location’ includes eye, head and neck, breast, perineum and genitalia

**c:** ‘Other referral route’ includes family member, former patient and self.

Across the study period 194 (21.8%) patients took CR8. Compared to the previous SR8 regimen, use of the CR8 regimen was strongly associated with treatment completion, with 93.4% (170/182) of those taking CR8 completing treatment compared to 80.7% (385/477) of those taking SR8 (OR 4.27, 95% CI 2.20–8.31, p <0.0001). We also found that occupation was associated with treatment completion, with pupils being more likely to complete treatment than farmers (OR 5.14, 95% CI 1.78–14.89, p = 0.005) (Table 2).

### Multivariable logistic regression

In the multivariable regression model (Table 3) there remained no statistically significant association between distance to clinic and treatment completion (10km+ OR 1.94, 95% CI 0.86–4.38, p = 0.111; 20km+ OR 1.25, 95% CI 0.57–2.76, p = 0.576). Due to the degree of missing data for this variable, the model appeared to suffer from sparse data and so a reduced model excluding distance to clinic was produced (Table 4). In both the model including distance (Table 3) and the reduced model, the CR8 treatment regimen was associated with higher treatment completion compared to use of the SR8 regimen (OR 4.1, 95% CI 2.03–8.27, p <0.001) (Table 4). There was no evidence of statistically significant associations between age, sex, referral route, use of traditional treatment or occupation and treatment completion in the final multivariable regression model.

**Table 3:** Basic and fully adjusted multivariable regression models for the association between exposure variables and treatment completion (including ‘distance to clinic’).

Variable	Basic model Adjusted* OR (95% CI) n = 363	P value**	Full model Adjusted* OR (95% CI) n = 325	P value**
**Sex**				
Female Male	1		1	
	1.03 (0.56–1.89)	0.924	0.85 (0.44–1.64)	0.621
**Age** (years)				
Per 1-year increase	0.98 (0.96–0.99)	0.004	1.00 (0.98–1.03)	0.773
**Distance to clinic** (km)				
0- 10- 20+	1		1	
	1.68 (0.79–3.54)	0.176	1.94 (0.86–4.38)	0.111
	1.64 (0.78–3.44)	0.189	1.25 (0.57–2.76)	0.576
**Treatment regimen**				
SR8 CR8	1		1	
	2.98 (1.20–7.40)	0.018	2.98 (1.18–7.54)	0.021
**Referral route**				
Health worker Village health volunteer Other			1	
			0.75 (0.36–1.59)	0.455
			1.08 (0.46–2.50)	0.862
**Occupation**				
Farmer			1	
Pupil			3.97 (1.40–11.24)	0.010
Other			1.80 (0.65–5.04)	0.261
**Traditional treatment use**				
No			1	
Yes			2.34 (1.03–5.35)	0.043

*Adjusted for all other variables in the column

**P values are based on the Wald test

**Table 4:** Basic and fully adjusted reduced multivariable regression models for the association between exposure variables and treatment completion (excluding ‘distance to clinic’).

Variable	Basic model Adjusted* OR (95% CI) n = 619	P value**	Full model Adjusted* OR (95% CI) n = 554	P value**
**Sex**				
Female Male	1		1	
	0.87 (0.55–1.36)	0.537	0.89 (0.55–1.44)	0.641
**Age (years)**				
Per 1-year increase	0.97 (0.96–0.99)	<0.001	0.98 (0.96–1.00)	0.098
**Treatment regimen**				
SR8 CR8	1		1	
	3.81 (1.94–7.51)	<0.001	4.10 (2.03–8.27)	<0.001
**Referral route**				
Health worker Village health volunteer Other			1	
			0.87 (0.51–1.50)	0.623
			1.38 (0.73–2.61)	0.321
**Occupation**				
Farmer			1	
Pupil			1.83 (0.83–4.03)	0.133
Other			2.05 (0.87–4.82)	0.102
**Traditional treatment use**				
No			1	
Yes			1.38 (0.81–2.36)	0.238

*Adjusted for all other variables in the column

**P values are based on the Wald test

## Discussion

In our current study we found the rate of treatment completion was 84.4% across four major BU clinics in the Ashanti and Central Regions between 2006 and 2018. The rate of treatment completion documented was much higher than the 46% found by Klis et al in the same region in 2014 [10] though there is still scope for improvement. We found clear evidence that the new fully oral CR8 regimen was associated with improved treatment completion. This is the first study to demonstrate that recent changes to recommend CR8 as first line treatment [1] might translate into improved treatment completion rates. Patients receiving fully oral therapy are able to self-administer treatment under the supervision of Community Based Surveillance Volunteers (CBSV), who are present within each community in a Health District. CBSVs receive regular training by the health service to support patients by making home visits, monitoring pill counts and providing a communication link to the health service should any issues arise [20]. The improved completion rates seen may reflect both the less demanding nature of CR8 on patients’ time and resources and the improved tolerability of CR8 compared to daily injections as part of the SR8 regimen, whilst the use of CBSVs to monitor care in the community allows ongoing monitoring and adherence support, reducing the risk that individuals may default from care if they do not need to regularly attend a health facility [10]. As we do not have detailed data on reasons for non-completion, we are not able to be certain if tolerability, provision of care in the community or other factors are the major drivers of improved compliance seen with the CR8 regimen. With a higher proportion of missing data for treatment completion in those who took SR8, it is possible that this effect could be greater than indicated by our analysis.

We did not find any evidence that distance to clinic was associated with treatment completion. Other studies have clearly documented the impact that indirect costs, in particular those related to transportation may have on treatment seeking and adherence [10]. Whilst it would be anticipated that greater distances are associated with increasing barriers to care our measure of distance to clinic is almost certainly an imperfect measure as it cannot fully capture variations in the time needed for and cost of accessing care. We did note important associations with lesion type and category and distance to clinic. In particular, increasing distance from clinic was associated with presenting with ulcerated lesions, osteomyelitis and category II and III lesions. These findings support suggestions that patients may delay seeking appropriate care for BU due to direct and indirect costs of transport to and accessibility of BU clinics [17,18]. The increasing severity of lesions amongst patients living further away from clinics might also reflect less awareness of the condition in these communities or less strong active case finding programmes in these settings. As more severe forms are likely to be associated with worse outcomes in terms of disability and stigma our data support the need to consider further decentralisation of care, and in particular access to early identification and treatment in order to improve rates of early detection of the disease.

The benefits of community-based care have been widely recognised in the management of other mycobacterial infections with lengthy treatment regimens, such as TB and leprosy. Similar barriers to diagnosis and treatment have been cited, such as long distances to health facilities and transport and opportunity costs incurred from accessing treatment [30,31]. Community health workers and volunteers have been regarded as key in supporting a model of care that addresses such issues, undertaking a range of roles including community outreach to increase early detection of disease, direct observation of treatment and psychosocial support [30,32] and consideration should be given to their use in the management of BU. New portable diagnostic tests may help facilitate this [33] whilst the introduction of the CR8 regimen should further facilitate fully decentralised and fully community-based care.

Our study has a number of limitations. Most notably we relied on routine clinical records and data was therefore missing for some variables. Whilst we attempted to minimise this by utilising both the records held at KCCR and the primary clinic copy of the BU01 form, missing data remains a challenge. Secondly, as already outlined there are a number of issues with the measurement of ‘distance to care’ [34]. Village names on BU01 forms could not always be matched with the spatial data [29] due to gaps in the spatial dataset, multiple villages within the region sharing the same name and the legibility of handwritten clinical notes. There was also difficulty with consistency in defining distance to care [34], as documented village of residence may not always reflect where the patient travelled from during treatment, for example with some staying with relatives closer to the clinic or hospitalised during treatment. Finally, point distance measurements do not fully consider clinic accessibility, not capturing the full length of the route, availability of public transport, the road conditions and the time and cost necessary for the journey. Despite this limitation we found clear evidence that increasing distance is associated with later presentation highlighting the need for ongoing efforts to improve early access to diagnosis.

There are also potential differences in the recording of treatment completion between regimens. Completed treatment was documented with differing frequency for SR8, measured through direct daily observation, compared to CR8 which was measured by fortnightly blister pack counts. Whilst this may have introduced some potential for bias, we believe it is unlikely to undermine the strong and highly plausible association seen between regimens and treatment completion. Finally, we had limited information available on potential confounders such as socioeconomic status. Future prospective studies, measuring both socio-economic variables and more accurately assessing access to care would greatly enhance our understanding of how to optimise BU treatment seeking behaviours and treatment completion.

Our study demonstrates that BU treatment completion in the Ashanti and Central Regions, Ghana are substantially higher with the now recommended oral CR8 regimen compared to the previous SR8 regimen. This finding, which likely reflects the benefits of both treatment tolerability and accessibility, are supportive of the notion of further decentralisation of BU care towards a fully community-based model. Further research is needed, to ascertain the most appropriate means of further decentralising care, for example self, peer or CBSV delivered wound care. We found evidence that distance to health facilities continues to be associated with more severe forms of BU. Any decentralised system must also therefore continue to support community engagement and active outreach to identify cases early, when they can be most easily managed in the community and when long term disability can be avoided. Ideally such a strategy would reduce costs both to patients, by removing costs of seeking care and to health systems, by detecting cases early when care and its associated costs are lower. The combination of point-of-care tests and oral treatment regimens makes such a strategy feasible, but investment and research will be needed to make it a reality.

## Supporting information

S1 Data Original data included for analysis. (XLSX)
